# Effects of Exercise on Quality of Life in Subjects with Alzheimer’s Disease: Systematic Review with Meta-Analysis of Randomized Clinical Trials

**DOI:** 10.3390/sports11080149

**Published:** 2023-08-09

**Authors:** Mariana Mendes, Érica Correia, Anabela Vitorino, José Rodrigues, Luís Cid, Teresa Bento, Raul Antunes, Diogo Monteiro, Nuno Couto

**Affiliations:** 1 Sport Sciences School of Rio Maior, Polytechnic of Santarém (ESDRM), 2040-413 Rio Maior, Portugal; mariana95mendes@hotmail.com (M.M.); ericamelissa31@hotmail.com (É.C.); anabelav@esdrm.ipsantarem.pt (A.V.); jrodrigues@esdrm.ipsantarem.pt (J.R.); luiscid@esdrm.ipsantarem.pt (L.C.); teresabento@esdrm.ipsantarem.pt (T.B.); ncouto@esdrm.ipsantarem.pt (N.C.); 2 Research Center in Sports Sciences, Health Sciences and Human Development (CIDESD), 5001-801 Vila Real, Portugal; diogo.monteiro@ipleiria.pt; 3 Life Quality Research Center (CIEQV), 2040-413 Rio Maior, Portugal; 4 ESECS, Polytechnic of Leiria, 2411-901 Leiria, Portugal; 5 Center for Innovative Care and Health Technology (ciTechCare), Polytechnic of Leiria, 2411-901 Leiria, Portugal

**Keywords:** dementia, interventions, physical activity, quality of life

## Abstract

Alzheimer’s disease is a type of dementia that progressively affects memory, thinking, and behavior. It can interfere with daily activities and lead to a decline in cognitive abilities over time. Exercise-based interventions can complement Alzheimer’s disease treatment. Exercise is a valuable tool in all healthcare settings and has shown promise as an effective cognitive improvement intervention for people with cognitive impairments. This systematic review and meta-analysis aimed to analyze the effect of physical exercise on the QoL of subjects with Alzheimer’s disease. A search was performed with the help of the electronic databases PubMed and Web of Science. Randomized controlled trials with exercise-based interventions were selected. Four studies met the inclusion criteria, which included interventions based on exercise. The effects were summarized using standardized mean differences (95% confidence intervals) using random-effect models. The results showed that exercise had no significant effect on the QoL of subjects with Alzheimer. Thus, the effect of exercise interventions on the QoL of patients with Alzheimer’s disease is not conclusive. More research is needed about this topic and the way in which the QoL is assessed; the necessity to conduct an objective way to assess the QoL in this population is mandatory.

## 1. Introduction

Alzheimer’s disease (AD) is the most common cause of dementia in subjects and has no treatment [1]. AD is as a primary degenerative disease of the brain and involves a gradual deterioration of cognitive abilities, including memory, thinking, comprehension, calculation, language, learning ability, and judgement, and can be expressed in three different categories or types: early-onset AD, late-onset AD, or sporadic AD and familial [2].

Alzheimer’s disease is a type of dementia that affects memory, thinking, and behavior. It is a progressive disease that interferes with daily activities and can lead to a decline in cognitive abilities. Apart from Alzheimer’s disease, there are various other types of dementia such as vascular dementia, dementia with Lewy bodies, and fronto-temporal dementia. Dementia can also occur following a stroke or in the presence of certain infections such as HIV, as well as due to the harmful use of alcohol or repetitive physical injuries to the brain (i.e., chronic traumatic encephalopathy) or nutritional deficiencies (Global Action Plan on the Public Health Response to Dementia 2017–2025) [3].

AD often leads to changes in a subject’s behavior, personality, and functional ability [4]. As the disease progresses, these subjects become increasingly dependent on others, and they end up needing care at the level of the elementary activities of daily living, such as personal hygiene, food, and clothing [4], which leads to a major impact on the quality of life, both for individuals living with dementia and for their families and caregivers [5].

According to Zhu et al. [6], recent epidemiological studies have shown that engaging in activities, such as bilingualism/multilingualism, education, occupation, musical experience, exercise, and leisure activities, may help to slow down the rate of memory loss and delay the onset of mild cognitive impairment (MCI) and dementia. These activities can stimulate brain function, improve the interaction between genes and the environment, increase cognitive repair and reserve, and delay brain aging [6].

According to the 2022 AD annual report [5], there has been a significant increase in deaths from AD and all forms of dementia in the United States in 2020 compared to the average of the previous five years. An estimated 6.5 million Americans are living with AD today. This number could grow to 13.8 million by 2060, barring the development of medical breakthroughs to prevent, slow, or cure AD. Official death certificates recorded 121,499 deaths from AD in 2019, which is the latest year for which data are available. AD was officially listed as the sixth-leading cause of death in the United States in 2019. Preliminary reports for 2021 show at least 11,000 more deaths from AD and other dementias compared to the average for the five years prior to 2020 [7].

Currently, treatment for AD is based on pharmacological drugs [8]. Several authors state that exercise-based interventions can be a non-pharmacological complement to AD treatment. In addition to health and wellness, exercise is a worthy tool in all health care settings. Furthermore, exercise has shown promise as an effective cognitive improvement intervention for older adults with cognitive impairments [9,10,11,12].

The WHO Report 2022 [13] reports that around 7–8% of all cases of cardiovascular disease, depression, and dementia could be prevented if subjects were more physically active.

Exercise can positively influence cardiovascular, hormonal, neurological, and respiratory levels [14]. Stimulating exercise thus appears to improve brain vitality and may be an intervention to reduce dementia-related decline [14] and improve the quality of life (QoL) [14].

The definition of QoL from the WHO is particularly illustrative of this type of approach: the QoL is defined therein as “an individual’s perception of his or her position in life in the context of his or her culture and value system, in relation to his or her goals, expectations, norms, and concerns [15]. It is a broad concept, influenced in a complex way by an individual’s physical health, psychological condition, level of autonomy, social relationships, personal beliefs, and their relationship with significant aspects of their environment” [15]. Instruments that are more commonly used in research to measure the QoL include the Quality of Life—Alzheimer’s Disease (QoL-AD) [16] and the European Quality of Life (EQ- 5D). Studies suggest that evaluating the QoL in individuals with dementia poses a challenge for both clinicians and researchers when it comes to measuring it accurately [17]; with this in mind, one can speculate if this is a reason why the QoL is not considered to be a primary variable in most studies with people with AD, and, therefore, more in-depth information should be gathered on the studies that objectively measured the QoL.

The numerous research works dedicated to analyzing the effects of exercise on people suffering from Alzheimer’s’ disease have been published in the last five years, which have also resulted in an high number of synthesis studies on the subject [16,17,18,19,20,21]. However, the results are still unclear about the effects of exercise in individuals with AD. On the other hand, the QoL has been assessed in a very limited number of studies, mainly as secondary outcome [22,23] or referred to as an indirect effect of exercise. As exercise has the potential to alleviate some symptoms of dementia, researchers assume that there is an improvement in the quality of life, however, the QoL was not assessed in most these studies [16].

As stated by López-Ortiz [21], previous meta-analyses analyzed the effects of exercise on AD; however, primary studies included, in these meta-analyses, selected randomized controlled trials (RCTs) and non-RCTs, which involved patients with dementias other than AD and combined exercise with other therapies, thereby inducing a high risk of bias. Also, López-Ortiz [21] and his colleagues failed to analyzed the effects of exercise in the QoL [21]. Camara-Calmaestra et al. [16] conducted a very recent systematic review of randomized controlled trials in order to evaluate the effectiveness of exercise on a series of AD-relevant measures, including the QoL. Results showed moderate evidence of the positive impact that exercise could have in improving the QoL in patients with AD disease; however, the results were narrowed to aerobic exercise [16].

For these reasons, the aim of the present systematic review and meta-analysis is to assess the effects of exercise on the directly measured quality of life of subjects with AD.

## 2. Materials and Methods

The present study followed the PRISMA (Preferred Reporting Items for Systematic Reviews and Meta-Analyses) guidelines [24]. According to what is recommended by the PRISMA protocol [24], an exploratory search was carried out to identify the most significant descriptors and databases for conducting our study. With this in mind, the search was conducted between May and October of 2022 using the U.S. National Library of Medicine’s Medical Subject Headings terms related to Alzheimer (#1) Exercise (#2), and Quality of Life (#3) (#1 And #2 And #3) in PubMed and WOS, with in all fields in English, with no restriction on the date of publication or descriptors. Potentially relevant articles were searched in the reference lists of the manuscripts obtained in the search, and other systematic reviews and meta-analyses were included if they contained relevant data. The present study was registered in the PROSPERO database under the number CRD42023398107.

### 2.1. Eligibility Criteria

The eligibility criteria of the studies were established according to the PICOS (Population, Intervention, Comparison, Outcomes, and Study Design) strategy, which is defined as follows:

Population: participants aged 18 years old or older, diagnosed with Alzheimer’s disease according to the criteria of a mild to moderate AD according to the criteria of the revised version of the DSM (Diagnostic and Statistical Manual) [25];
Intervention: interventions based on exercise;
Comparison: Alzheimer’s diseases participants who maintained their daily activities with standard care for Alzheimer disease;
Outcomes: quality of life;
Type of study: randomized controlled trial (RCT);

Studies were excluded if they comprised the following: (1) included participants with age below 18 years old; (2) integrated interventions other than exercise; (3) lacked comparison between intervention group (IG) and control group (CG) results; (4) comprised description of exercise program characteristics that was unclear; (5) included participants with associated diseases or physical dependence; (6) studies were not written in English; and (7) consisted of non-original articles such letters to editors, trial registrations, proposals for protocols, editorials, book chapters, and conference abstracts.

### 2.2. Study Identification

An initial screening was conducted based on titles and abstracts, followed by selection through reading of the full text of the manuscripts. The search was carried out independently by two researchers between independently. In case of conflicts, an additional element was included to achieve a final decision on the inclusion or exclusion of RCTs. Finally, all studies were read in full to obtain the final selection of studies.

### 2.3. Data Extraction

The following data was extracted from studies: country of origin, authors, design, number of participants, age, gender, type of exercise, intensity, outcomes, and conclusions of the study.

### 2.4. Quality of Study and Risk of Bias

We used the risk of bias tool to assess the QoL studies used. The risk of bias was classified as low risk, unclear/unknown, or high risk. We used seven types of criteria for bias: random sequence generation (selection bias), allocation concealment (selection bias), blinding of participants and personnel (performance bias), blinding of outcome assessment (detection bias), incomplete outcome data (attrition bias), selective reporting (reporting bias), and other bias [26].

### 2.5. Data Synthesis and Analysis

Meta-analyses were performed for studies that compared exercise interventions using the Cochrane Review Manager Software (RevMan 5.4.1). The standard mean difference (SMD) of QoL measurements pre- and post-intervention were calculated. The standard deviation (SD) of the mean difference, when not presented in the studies, was estimated using procedures recommended by the Cochrane handbook [27]. Heterogeneity was analyzed using the statistics of Chi^2^ and *I*^2^, where a value of *p* > 50% indicated considerable heterogeneity [28].

## 3. Results

### 3.1. Results of the Systematic Literature Search

**Figure 1:**
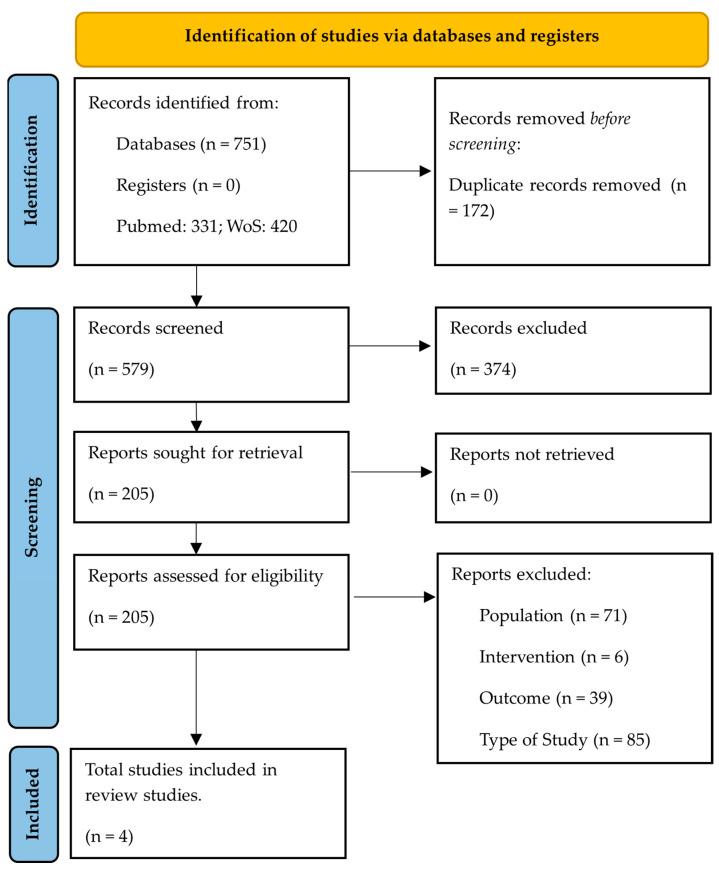
Flowchart of included and excluded articles according to PRISMA.

A total of 751articles were identified. After screening, 201 studies were excluded based on inclusion and exclusion criteria, which resulted as follows: outcome (n = 39), study type (n = 85), different populations (n = 71), and different interventions (n = 6). The details of the included and excluded studies are shown in the flow chart [24] in Figure 1.

### 3.2. Study Characteristics

Table 1 shows the characteristics of each included study regarding the country, participants, outcomes, and type of intervention. The included studies were conducted in several countries, such as the United States, China, and France. A total of 716 people participated, with 640 completing the intervention. The age range was between 60 and 96 years old. Two questionnaires were used to assess the participants’ quality of life, namely, the EuroQol (assesses the quality of life in health) and the Qol-AD (assesses the quality of life in Alzheimer’s patients); three tools were also used to assess the participants’ cognitive levels: the ADAS Questionnaire—Cog (Alzheimer’s Disease Cognitive Assessment Scale) [29,30], the MoCA Questionnaire (assesses patients’ cognitive health) [31], and the MMSE (Minimum Mental State Examination) Questionnaire [29,30].

The type of intervention performed in all studies (n = 4) was aerobic training for the intervention group and standard care for the control group, and one study included combined training, including strength training. Relative to the intervention period, the minimum period was nine weeks [22], compared to the maximum period of 12 months [29]. The frequency of each session ranged from two [22,29] to three [29,31] times per week, and the duration ranged from 25 min [29] to 60 min per session [31].

Strength training consisted of arm exercises using weights, front lifts, side lifts, and leg strength exercises [29]. The starting weight ranged from 0 to 12 kg, depending on ability. The baseline goal for the strength training exercises was three sets of 20 repetitions. The sets had to be at least moderate to intense intensity, and the weight was increased accordingly. In subsequent sessions, weight amounts were increased to ensure progression, with decreased repetitions if necessary [29].

**Figure 2:**
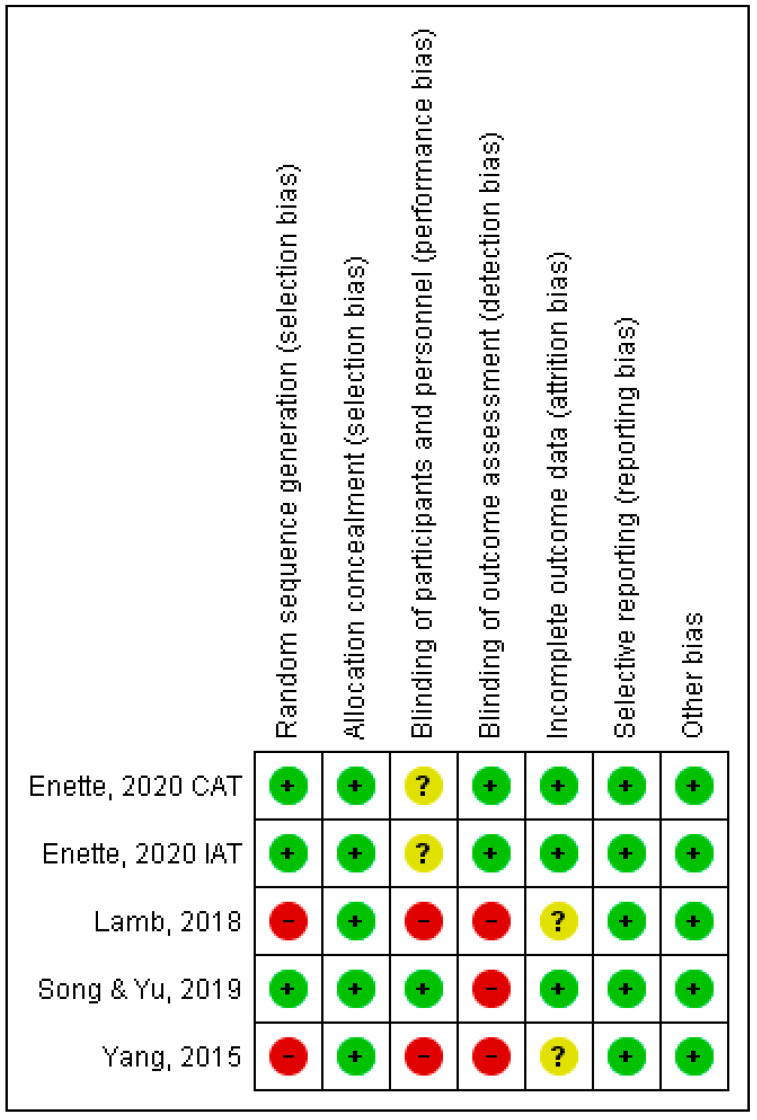
Summary of the risk of bias for each article included [22,29,30,31] in the study (Bias).

Regarding the risk of bias, as shown in Figure 2, the study by Enette et al. [22] presented only uncertain risk in the way participants were selected (blinding of participants). The study by Song and Yu [31] showed a low risk of bias in all categories except for the way the outcome was presented (blinding of outcome), which showed a high risk of bias. The other two studies [29,30] showed a high risk of bias, namely in the categories (random generation, blinding of participants, and blinding of outcome).

**Figure 3:**
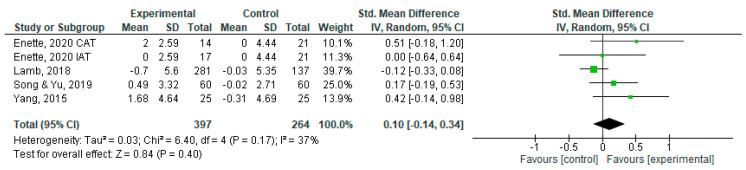
Forest plots showing the effects of exercise on QoL outcome [22,29,30,31]. Note: SD—standard deviation; CI—confidence interval; IV—inverse variance.

Finally, as shown in Figure 3, we can observe that exercise had no significant effect on QoL-AD results (SMD = 0.10; 95% CI, −0.14 to 0.34; *p* > 0.001).

## 4. Discussion

This systematic review with meta-analysis aimed to analyze the effects of exercise on the QoL of subjects with AD.

Our initial search, performed on PubMed and Web of Science databases, found 751 studies. After screening, only four studies meet our inclusion criteria and were included in our analysis. Regarding the included studies, overall, there was no significant effect in favor of exercise intervention, and, individually, each study did not achieve any significant effect. As for the type of exercise used, we observed that studies that evaluated aerobic exercise alone [31,32] had a more significant effect related to the QoL than studies that combined aerobic and strength exercise [29].

The Enette et al. (2020) study, which consisted of two intervention groups who partook in continuous aerobic training (CAT) and interval aerobic training (IAT) and one control, concluded that the CAT group achieved significant improvements in the QoL compared to the IAT group and the control group. The type of intervention used was aerobic exercise lasting 30 min per session for two times per week [22].

When analyzing the Song and Yu [31] study, the intervention group achieved significant improvements in health related the QoL compared to the control group. The type of intervention was aerobic exercise, which lasted 60 min for three times per week [31]. This study also concluded that, while the control group showed deterioration in cognitive function over time, the subjects who were engaged in regular exercise (i.e., the intervention group) had a significant improvement in the same health parameter. This finding implies that moderate intensity aerobic training can provide the treatment goal of preventing deterioration among older subjects with MCI [31]. Based on various authors, it seems clear that CAT is beneficial to AD subjects [22,32,33]. According to Enette et al. [22], by participating in regular aerobic exercises, individuals may experience improvements in their social relationships and emotional well-being, and CAT may have more of an impact on overall well-being in this population. Considering the intensity of exercise, moderate intensity also seems to be more beneficial to this population than high intensity [22]. Moderate-intensity exercise has been found to be effective in improving cognitive function and neuropsychiatric symptoms in AD subjects, wherein it promotes blood flow, increases hippocampal volume, and stimulates neurogenesis, which are all beneficial for individuals with AD [23,34]. Otherwise, high-intensity exercise may cause excessive stress and fatigue, thereby potentially exacerbating cognitive decline and neuropsychiatric symptoms [23]. Moreover, moderate-intensity exercise allows individuals with AD to engage in social interactions and participate in group activities, which can enhance their overall well-being and quality of life [34]. The study of Lamb et al. [29] corroborates the previous statement, wherein their study concluded that a four-month moderate- to high-intensity aerobic and strength exercise program, when added to usual care, does not slow cognitive decline in subjects with mild to moderate dementia. Exercise improved short-term fitness, but this did not translate into improvements in the activities of daily living, behavioral outcomes, or health-related QoL.

The results from Yang et al. [30] tell us that the results of the aerobic group showed increased MMSE and Qol-AD scores after three months of study. However, it was also noted that the Qol-AD scores did not obtain significant differences [30].

The type of intervention varied from study to study, and the intervention period ranged from at least nine weeks [22] to the maximum period lasting 52 weeks [29]. The interventions had a weekly frequency between two [22,29] to three [30,31] times per week, and the duration ranged from 25 [30] to 90 [29] minutes per session. International guidelines recommend that older adults with Alzheimer’s disease should engage in moderate-intensity aerobic exercise for at least 150 min per week, or 30 min per day, five days per week, and resistance training should also be included at least twice per week [35]. None of the included interventions achieved the international recommendations of exercise for this population. In addition, since the type of exercise differed greatly between studies, it was impossible to draw any further conclusions on the effect of exercise on AD.

Liu et al. [36], when analyzing tests that measured cognitive performance, found that the MMSE test and ADAS—Cog showed significant improvements in cognitive performance, while studies using the MoCA test showed no significant improvements. Regarding our review, both studies that used the MMSE and ADAS—Cog, as well as the MoCA, obtained a positive, but not significant, effect on the QoL of these subjects.

Research suggest that evaluating the QoL in individuals with dementia poses a challenge for both clinicians and researchers when it comes to measuring it accurately [17]. Several studies have found significant discrepancies between the perceptions of individuals with dementia and their caregivers regarding their QoL.

The theoretical construct of the QoL in dementia involves the self-appraisal of their internal (subjective) and external (objectionable) reality, its interpretation and integration, and considering their beliefs and values, and this process is strongly influenced by mood [37,38,39]. This conceptualization assumes that there are aspects of the QoL that can only be assessed by the subject him/herself, so it is expected that there will be a difference in the interpretation of the QoL by the subject and his/her caregiver [37,38,39].

It is often observed that higher QoL ratings by caregivers are influenced by lower levels of dependency in subjects with dementia [40]. Studies investigating the caregiver and individual perceptions of QoL scores were strongly affected by the individual’s mood and caregiver experience [41,42,43]. These studies explored subjects with mild to moderate dementia living in the community and found that lower QoL ratings of the subjects with dementia were predicted by the presence of depressive symptoms, while lower caregiver ratings were associated with depression and caregiver burden [40].

On the other hand, it should be noted that cognitive impairment might influence how individuals with advanced dementia perceive and assess their QoL. Moreover, these factors could limit the effectiveness of interventions, such as exercise initiatives aimed at enhancing the quality of life for individuals with dementia, as has been indicated by previous research [17].

Consistent with these limitations, it is regularly the case that subjects with dementia rate their QoL better than the ratings by their caregivers or other observers [40,44,45].

An approach that includes a combination of self-report and a physical examination report may provide more complete information about the QoL in subjects with dementia [36].

## 5. Conclusions

Through this systematic review and meta-analysis, where we analyzed the effect of exercise on the QoL in Alzheimer’s subjects, it was possible to verify that exercise interventions had no significant effect on the quality of life in subjects with AD. Since the type of exercise differed greatly between the studies, it was impossible to draw any further conclusions regarding the effect of exercise on AD.

Based on the literature, exercise is important and necessary in the general population. Therefore, it remains necessary to understand how exercise could promote the QoL in these subjects. Further research on this topic will be necessary so that we can help improve the QoL of these subjects. According to the characteristics of this disease, more research is needed on the topic, and assessments of the QoL should rely on objective measures in this population.

## Figures and Tables

**Table 1:** Characteristics of the included studies.

Study	Country	Participants	Age (Years) (M ± SD)	Outcomes	Type of Intervention	Conclusions
**Lamb et al., 2018** [29]	United States	Intervention group n = 281 (men:166/women:112); Control group n = 137 (men: 86/women: 51)	76.9 (7.9)	ADAS—Cog; Qol-AD; EuroQol	Aerobic exercise, 25 min of moderate- to hard-intensity cycling, depending on tolerance level, and strength exercise, three sets of 20 repetitions at gym; duration 60 to 90 min per session; 2x per week; one hour of home exercises per week; 16 weeks.	Exercise improved short-term physical fitness, but this did not translate into improvements in health-related quality of life.
**Song & Yu, 2019** [31]	China	Intervention group n = 60 (men: 48/women: 12; Control group n = 60 (men: 42/women:18)	75.78 (6.28)	MoCa; Qol-AD	Aerobic Exercise; moderate intensity; duration 60 min; 3x per week; 16 weeks	Participants in IG had a significant improvement in health-related quality of life compared to CG.
**Yang et al., 2015** [30]	China	Intervention Group n = 25 (men: 10/women:15); Control Group n = 25 (men:7/women:18)	72.5 (10.6)	ADAS-Cog; MMSE; Qol-AD	Aerobic Exercise; initial time 25 to 30 min and after 1 week increased to 40 min; 3x per week; 12 weeks	Aerobic exercise could improve cognitive function, mental status, and quality of life in AD patients.
**Enette et al., 2020** [22]	France	Intervention group n = 31 (CAT = 14 (men:3/women:11)/IAT = 17(men:6/women:11); Control group n = 21 (men:10/women:11)	79 (24)	MMSE; Qol-AD	Aerobic Exercise; duration 30 min; 2x per week; 9 weeks	In the CAT group, there were improvements in quality of life compared to the other two groups (in mood and financially).

Note: M—mean; SD—standard deviation; CAT—continous aerobic training; IAT—interval aerobic training; MMSE—Minimum Mental State Examination; Qol-AD—Quality of Life in Alzheimer’s Patients; ADAS—Cog—Alzheimer ’s Disease Assessment Scale—Cognition; MoCa—Montreal Cognitive Assessment; EuroQoL—Health-Related Quality of Life; IG—intervention group; CG—control group.

## Data Availability

Additional data are available upon request to the author for correspondence.
